# Transcatheter hepatic arterial chemoembolization and sorafenib for hepatocellular carcinoma: a meta-analysis of randomized, double-blind controlled trials

**DOI:** 10.18632/oncotarget.19334

**Published:** 2017-07-18

**Authors:** Jun Li, Wenhui Liu, Wenhua Zhu, Yinqiao Wu, Benyan Wu

**Affiliations:** ^1^ Department of Gastroenterology, Chinese PLA General Hospital, Beijing 100853, China; ^2^ Department of Oncology, Chinese 309th Hospital of PLA, Beijing 100091, China

**Keywords:** randomized controlled trial, transcatheter hepatic arterial chemoembolization, hepatocellular carcinoma, disease progression, overall survival

## Abstract

We performed a meta-analysis of transcatheter hepatic arterial chemoembolization (TACE) combined with sorafenib for hepatocellular carcinoma (HCC), which included 4 double-blind, randomized controlled trials (RCTs) that investigated the effects of TACE combined with sorafenib (experimental groups) on time to disease progression (TTP), overall survival (OS), and various sorafenib-related adverse events, compared to those in the placebo (control) groups. A total of 877 HCC cases from 14 countries, including China and the USA, were included in our meta-analysis. The TTP increased significantly in the experimental groups (hazard ration [HR]: 0.82; 95% CI: 0.69–0.97; *p* = 0.02), but OS did not improve significantly (HR: 0.97; 95% CI: 0.72–1.29; *p* = 0.82), compared with the control groups. The risks of hand and foot skin reactions (HFSR), rash, fatigue, and diarrhea were significantly greater in the experimental groups (*p* < 0.05 for all), compared to those in the control groups, whereas the risk of nausea was statistically similar (*p* > 0.05). Among these, the risk of HFSR was highest (risk ratio [RR]: 5.93; 95% CI: 2.00–17.53; *p* = 0.001), and a subgroup analysis of studies that lacked significant heterogeneity in the HFSR data showed a higher risk of HFSR (RR: 10.96; 95% CI: 5.54–21.69; *p* < 0.05). In conclusion, although TACE plus sorafenib increases TTP, it does not improve OS. Therefore, the risk of the adverse events of TACE plus sorafenib should be considered as a potential therapeutic limitation.

## INTRODUCTION

Liver cancer is the second and sixth leading cause of cancer-related deaths worldwide among men and women, respectively [1]. Approximately 70% to 90% of primary liver malignancies are hepatocellular carcinoma (HCC) [2, 3]. A diagnosis of HCC is often made in patients with intermediate- to late-stage disease due to the mild, nonspecific symptoms associated with HCC onset [4], resulting in poor prognosis in most cases [3]. Therefore, radiotherapy and chemotherapy may be more effective than curative surgery for treating late-stage disease due to the invasive nature of HCC [5]. The combination of these factors make HCC a serious public health problem, especially in Eastern and Southeastern Asia [1].

Transcatheter arterial chemoembolization (TACE), which is used to temporarily block blood flow to tumor sites, has been shown to be effective for the treatment of HCC patients who are not candidates for surgical resection, and often causes fewer side effects than conventional systemic chemotherapy methods [6, 7]. However, the localized ischemia and hypoxia brought about during TACE treatment can activate tumorigenic VEGF, IGF-2, and bFGF signaling pathways in some tumors, which can subsequently stimulate angiogenesis and the growth of residual tumor cells, leading to tumor recurrence and metastasis [8, 9]. Therefore, the suppression of these precancer pathways during TACE treatment for HCC might improve clinical outcomes.

Sorafenib has been shown to suppress tumor growth and inhibit angiogenesis by inhibiting Raf kinase and receptor tyrosine kinases [10, 11], and has demonstrated relatively good therapeutic effects for the treatment of HCC [11, 12]. In recent years, researchers have evaluated TACE combined with sorafenib in the treatment of HCC, but the findings of clinical studies have been inconsistent, especially with regard to whether TACE combined with sorafenib [13, 14]. Two recent meta-analyses showed that TACE with sorafenib for HCC improved time to disease progression (TTP), but did not improve overall survival (OS), compared with that of TACE alone. However, 3 of the 5 studies included in those meta-analyses were not RCTs [13–17].

A meta-analysis is therefore needed to clarify the results of randomized controlled trials (RCTs) of TACE combined with sorafenib, thereby minimizing the potential effects of selection bias inherent in nonrandomized clinical studies. To this end, we performed a meta-analysis of the results of double-blind RCTs that investigated the effects of TACE combined with sorafenib in which TTP or OS were evaluated as primary endpoints and sorafenib-related adverse events were reported as secondary endpoints. Our results suggest that treatment using TACE with sorafenib does not provide significant benefit to HCC patients, compared with that of TACE alone, and that greater consideration should be placed on the adverse events of TACE plus sorafenib.

## MATERIALS AND METHODS

### Search strategy

This report was prepared according to the Preferred Reporting Items for Systematic Reviews and Meta-Analyses (Supplementary Table 1) guidelines [18]. The Institutional Review Board of our institution deemed our study to be exempt from review because only publicly available data were included in our analysis. We searched for the MEDLINE, EMBASE, EBSCO, Springer, Ovid, and Cochrane Library databases for published reports of RCTs published in English that evaluated the effects of transcatheter hepatic arterial chemoembolization combined with sorafenib on HCC clinical outcomes. The following keywords were used for our search: “transcatheter hepatic arterial chemoembolization”, “carcinoma, hepatocellular” [MeSH], “chemoembolization, therapeutic” [MeSH], “hepatic artery” [MeSH], “sorafenib” [MeSH], and “randomized controlled trial” [MeSH]. We used a date range for our search ending in 2016. The References sections of retrieved articles were also searched manually to identify other relevant RCTs.

### Selection criteria

The inclusion criteria for our meta-analysis were as follows: RCT; article was published in English; evaluated TACE combined with sorafenib for the treatment of HCC; patients were not suitable candidates for surgical resection; participants were 18 years of age or older; participant selection was not restricted based on sex; primary endpoints included TTP or overall survival (OS); and reported adverse events among patients receiving sorafenib as secondary endpoints. The exclusion criteria were as follows: Allocation not randomized; double blinding not performed; not placebo controlled; included patients with advanced HCC with significant vascular invasion or distant metastases; included patients diagnosed with another severe liver disease; included patients diagnosed with other types of malignant tumors, heart failure, renal failure, or human immunodeficiency virus infection.

### Data extraction and quality assessment

The following data were extracted: General information, including article title, authors’ names, and journal title; study characteristics, including study design, inclusion and exclusion criteria, study population size and demographic variables, duration of treatment, follow-up duration, and methods of bias prevention; clinical outcome measures (primary endpoints); data regarding loss to follow-up and exit status; adverse events among sorafenib users (secondary endpoints), including fatigue, diarrhea, nausea, hand and foot skin reactions, and/or other types of dermatitis. Quality assessment was performed based on the following criteria: Allocation was sufficiently randomized; blinding for allocation was sufficient; blinding for the intervention was sufficient; and loss to follow-up or exit status were evaluated.

### Statistical analysis

The statistical analysis was performed using the Rev.Man 5.2 software provided by Cochrane collaboration network. We used the *I*^2^ statistic to evaluate heterogeneity in the data, with *I*^2^ > 50% indicating significant heterogeneity. Effect size for the primary endpoints was evaluated based the hazard ratio (HR) and 95% confidence interval (CI) of TTP and OS. Effect size for the secondary endpoints was evaluated as the risk ratio (RR) and 95% CI based on the incidences of the various adverse events reported. Fixed-effects models were used to estimate risk when significant heterogeneity in the data was not detected (*I*^2^ ≤ 50%), whereas random-effects models were used when significant heterogeneity existed (*I*^2^ > 50%). The overall effect size was evaluated using the *Z* test, with a *P* < .05 indicating a statistically significant difference between the data for the experimental and control groups. Forest plots were constructed to represent the results of the risk analyses.

## RESULTS

### Study selection

**Figure 1:**
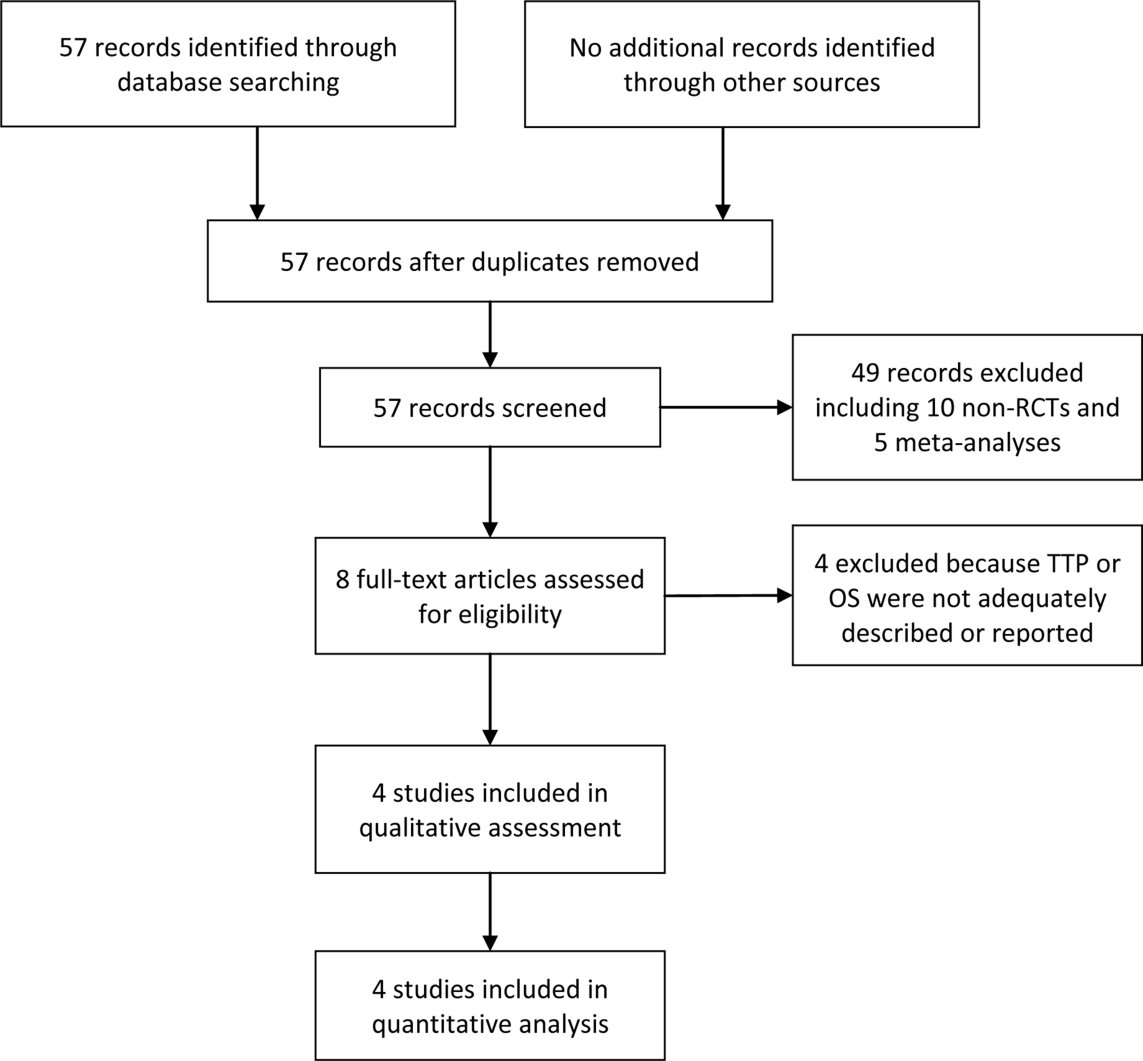
Flow diagram of study selection

As shown in the flow diagram in Figure 1, we initially retrieved 57 documents. Forty-nine of these articles were excluded based on the content of the title or abstract, among which 10 were non-RCTs of TACE combined with sorafenib for HCC and 5 were systematic reviews or meta-analyses. Four additional articles were excluded following the evaluation of full-text articles based on the inclusion and exclusion criteria for our study. The remaining 4 RCTs were included in our meta-analysis [19–22].

### Study characteristics

The characteristics of the selected RCTs are presented in Table 1. The studies were published between 2011 and 2016. Three of the studies were multicenter RCTs. The study locations included Australia, Austria, Germany, Spain, France, Belgium, Italy, Japan, Republic of Korea, Singapore, Taiwan, China, Canada, and the USA. The study populations included a total of 877 HCC cases who ranged in age from 58 to 73 years. All of the experimental groups were treated with TACE combined with sorafenib, and the control groups received TACE and placebo. The various studies used different methods of randomization for allocation to the experimental and control groups. The dosage of sorafenib was 400 mg twice daily in all of the studies. Blinding and randomization were determined to be adequate in all of the selected RCTs, whereas only one of these studies adequately described allocation concealment [21]. The various adverse events reported included fatigue, diarrhea, nausea, hand and foot skin reaction (HFSR), and other types of dermatological events referred to hereafter as rash. The risks of selection, performance, or detection biases were not quantified in any of the included studies.

**Table 1:** Study characteristics

Authors, year	Sorafenib treatment	TACE drug	Randomized	Blinding	Placebo controlled	Allocation concealed	Study Centers	Countries
Kudo et al., 2011	After 1 or 2 TACE sessions	epirubicin, cisplatin, doxorubicin, mitomycin	yes	Double	yes	no	4	1
Sansonno et al., 2012	30 days after TACE	Doxorubicin, mitomycin C	yes	Double	yes	yes	75	2
Hoffmann et al., 2015	Stopped 3 days before and resumed 3 days after TACE	Carboplatin	yes	Double	yes	no	85	13
Lencioni et al., 2016	3−7 days after first TACE	DEB-TACE, doxorubicin	yes	Double	yes	no	1	1

### Clinical outcomes

**Figure 2:**
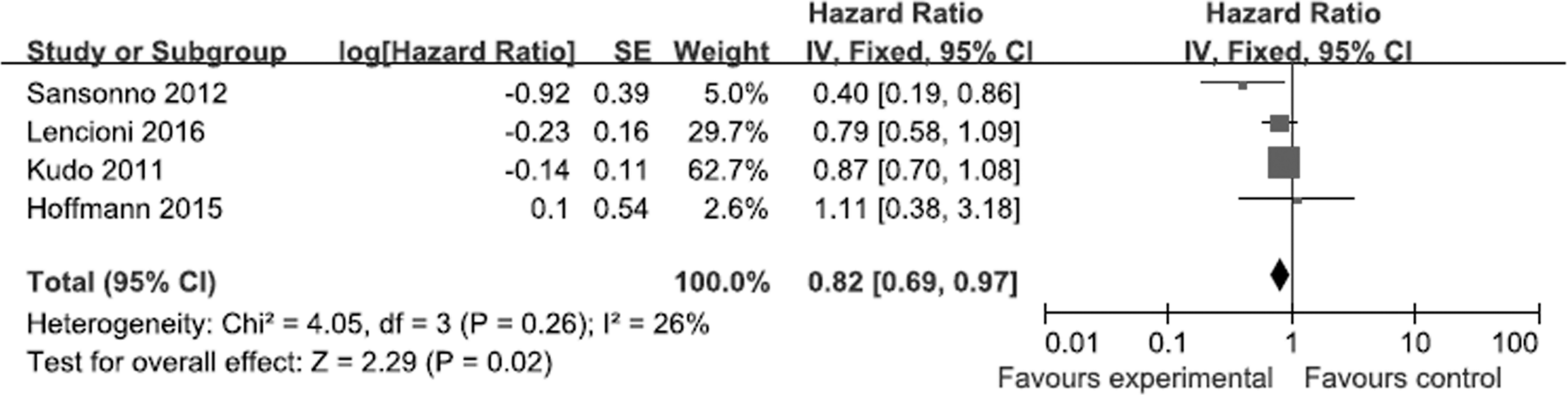
Comparison of time to disease progression Forest plots show squares representing the odds ratio for the experimental group compared with the placebo control group. Size of the squares is proportional to the size of the trials, and error bars represent the 95% confidence interval, with the diamond-shaped object representing the pooled estimates within each analysis.

**Figure 3:**
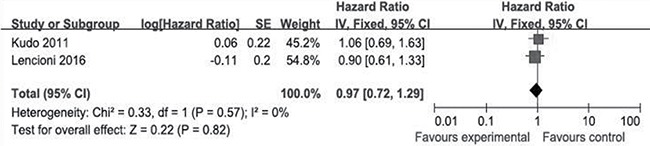
Comparison of overall survival Forest plots show squares representing the odds ratio for the experimental group compared with the placebo control group. Size of the squares is proportional to the size of the trials, and error bars represent the 95% confidence interval, with the diamond-shaped object representing the pooled estimates within each analysis.

As shown in Figure 2, all of the selected RCTs reported the median and 95% CI of TTP for the experimental and control groups. No selective reporting of clinical outcomes was observed. Significant heterogeneity in the TTP data was not observed (*I*^2^ = 26%). In our comparison of TTP between the experimental and control groups, the total HR of TTP was 0.82 (95% CI: 0.69–0.97; *Z* = 2.29; *p* = 0.02). The results suggest that the risk of disease progression in the experimental groups was lower than that in the control groups. Two of the selected RCTs [19, 22] reported the median and 95% CI of OS for the experimental and control groups (Figure 3), whereas the other selected RCTs did not report OS data [20, 21]. Significant heterogeneity in the OS data was not observed (*I*^2^ = 0%). In our comparison of OS between the experimental and control groups, the total HR of OS was 0.97 (95% CI: 0.72–1.29; *Z* = 0.22; *p* = 0.82). The results suggest that the survival of the patients in the experimental groups was not significantly different than that for patients in the control groups.

### Adverse events

### Risk of fatigue

**Figure 4:**
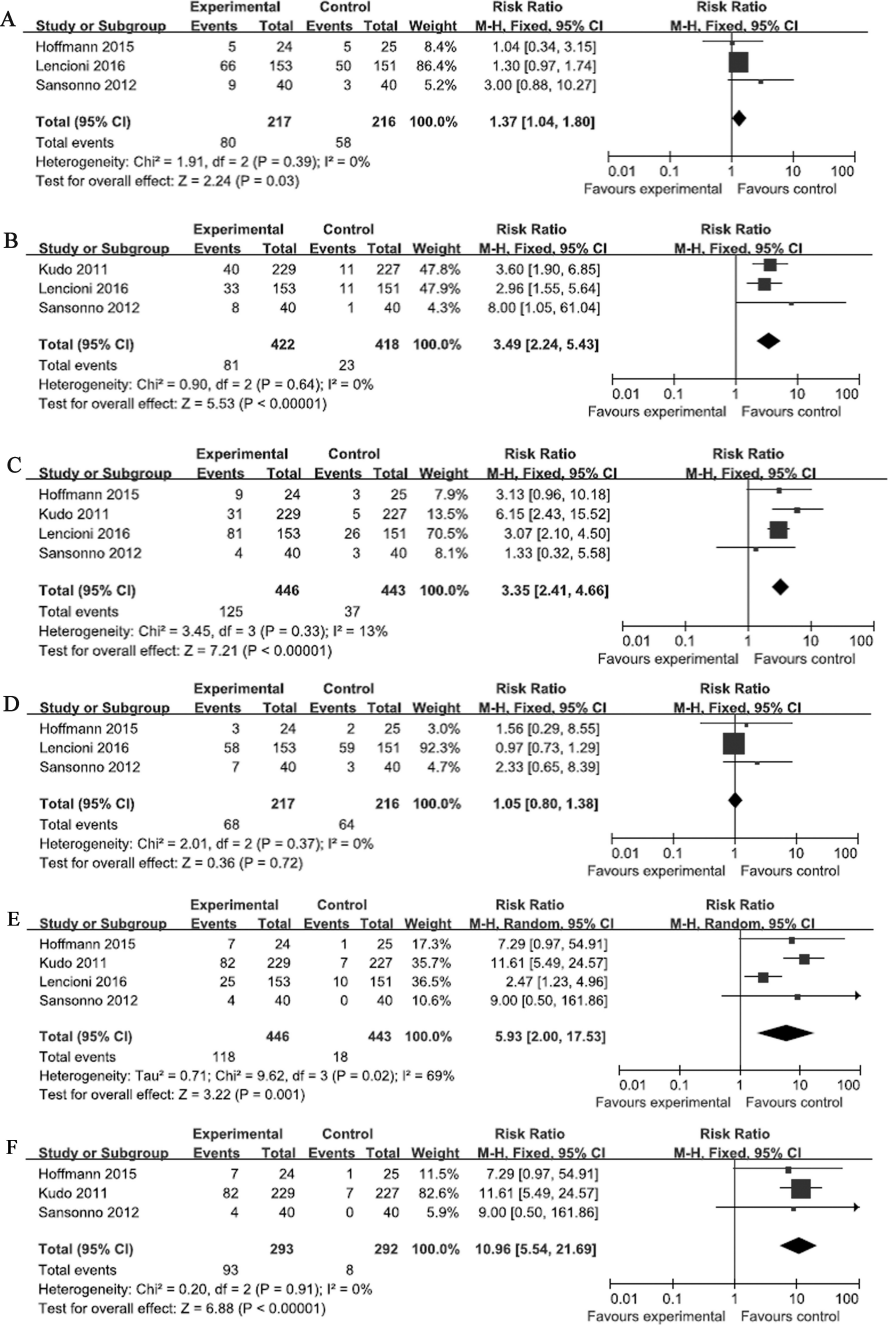
Comparisons of the risks of adverse events Forest plots depict the comparisons of the risks of (**A**) fatigue, (**B**) rash, (**C**) diarrhea, (**D**) nausea in the relevant studies, and the comparisons of hand and foot skin reactions in (**E**) all of the selected studies and (**F**) the studies in which drug-eluting beads were not used. Squares represent the odds ratio for the experimental group compared with the placebo control group, with the size of the square being proportional to the number of cases evaluated. Error bars represent the 95% confidence interval, and the diamond-shaped object representing the pooled estimates within each analysis.

Three of the selected RCTs compared the incidence of fatigue between the experimental and control groups [19–21]. No significant heterogeneity was observed in the fatigue data. The total RR of fatigue was 1.37 (95% CI: 1.04–1.80; *Z* = 2.24; *p* = 0.03; Figure 4A). These results suggest that the risk of fatigue among the patients in the experimental group was significantly higher than that among patients in the control groups.

### Risk of rash

Three of the selected RCTs compared the incidence of rash between the experimental and control groups [19, 21, 22]. No significant heterogeneity was observed in the rash data (*I*^2^ = 0%). The total RR of rash was 3.49 (95% CI, 2.24–5.43; *Z* = 5.53; *p* < 0.0001; Figure 4B). These results suggest that the risk of rash was significantly greater among the patients in the experimental groups than that in the patients in the control groups.

### Risk of diarrhea

Four of the selected RCTs compared the incidence of diarrhea between the experimental and control groups. No significant heterogeneity was observed in the rash data (*I*^2^ = 13%). The total RR of diarrhea was 3.35 (95% CI: 2.41–4.66; *Z* = 7.21; *p* < 0.05; Figure 4C). These results suggest that the risk of diarrhea was significantly greater among the patients in the experimental groups than that in the patients in the control groups.

### Risk of nausea

Three of the selected RCTs compared the incidence of nausea between the experimental and control groups [19–21]. No significant heterogeneity was observed in the nausea data (*I*^2^ = 0%). The total RR of nausea was 1.05 (95% CI: 0.80–1.38; *Z* = 0.36; *p* = 0.72; Figure 4D). These results suggest that the risk of nausea among the patients in the experimental groups was not significantly greater than that in the patients in the control groups.

### Risk of HFSR

All of the selected RCTs reported the incidence of HFSR in the experimental groups. Significant heterogeneity observed in the HFSR data (*I*^2^ = 69%). The total RR of HFSR was 5.93 (95% CI: 2.00–17.53; *Z* = 3.22; *p* = 0.001; Figure 4E), which suggested that the risk of HFSR among patients in the experimental groups was significantly greater than that of patients in the control groups. A subgroup analysis was also performed in which the results of the RCT that used drug-eluting beads for TACE [19] were excluded. No significant heterogeneity was observed in the HFSR data used in the subgroup analysis (*I*^2^ = 0%), and the total RR of HFSR was 10.96 (95% CI: 5.54–21.69; *Z* = 6.88; *p* < 0.05; Figure 4F). These results also suggest that the risk of HFSR among patients in the experimental groups was significantly greater than that of patients in the control groups.

## DISCUSSION

Sorafenib is the only anticancer drug that has been approved for systemic therapy for HCC [14, 23]. Studies have shown that sorafenib combined with doxorubicin was more effective for the treatment of intermediate- to late-stage HCC than doxorubicin alone [24, 25], and multiple RCTs have evaluated TACE plus sorafenib for late-stage or nonresectable HCC [19–22, 26]. Our meta-analysis of RCTs that investigated the use of TACE with sorafenib or placebo found that, although TACE combined with sorafenib significantly increased TTP (HR: 0.82; 95% CI: 0.69–0.97; *p* = 0.02), TACE with sorafenib did not improve OS significantly (HR: 0.97; 95% CI: 0.72–1.29; *p* = 0.82), compared to TACE plus placebo. Our findings suggest that other factors contribute to poor prognosis in HCC patients despite the delay in disease progression that is experienced in patients receiving TACE plus sorafenib.

Mild to moderate adverse events have been reported in patients treated with sorafenib [12, 24, 27], including HFSR, rash, fatigue, diarrhea, and nausea. Therefore, we also compared the incidence of these adverse events in patients receiving TACE plus sorafenib to those in patients receiving TACE plus placebo. Our meta-analysis showed that risks of HFSR, rash, fatigue, and diarrhea were significantly greater in the patients treated with TACE plus sorafenib (*p* < 0.05 for all), compared to those in patients treated with TACE plus placebo, whereas the risk of nausea was statistically similar in the experimental and control groups in the selected RCTs (*p* > 0.05).

A recent multicenter study conducted in China, Switzerland, and the USA reported that adverse dermatological events related to sorafenib use were associated with survival in intermediate-stage HCC patients treated with TACE and sorafenib in combination [28]. Our results showed that the increases in sorafenib-related HFSR and rash were high with RRs of 5.93 and 3.49, respectively. However, as noted above, median OS did not differ significantly between the TACE plus sorafenib and TACE plus placebo groups in the RCTs included in our meta-analysis. Although our analysis did not directly evaluate the prognostic value of HFSR and rash, our findings seem to be inconsistent with HFSR and rash being positively correlated with survival. Therefore, the prognostic value of sorafenib-related adverse dermatological events might be HCC-stage or time course dependent.

Our findings are, however, subject to certain limitations. We did not assess statistical power for our analysis, but the relatively small number of RCTs included in our meta-analysis represents a potential confounder of our findings. The small number of selected RCTs also precluded the use of funnel plots to assess whether publication bias influenced our findings, but the randomization and double blinding performed in all of the RCTs included in our meta-analysis likely minimized the potential effects of selection, performance, and detection biases. Nonetheless, significant heterogeneity in the TTP or OS data reported was not detected for the RCTs included in our analysis (*I*^2^: 26% and 0%, respectively). In addition, although significant heterogeneity was observed in the HFSR data (*I*^2^ = 69%), no significant heterogeneity was detected for the data used in the subgroup analysis of HFSR, and both the overall and subgroup results showed that the risk of HFSR was greater in the TACE plus sorafenib groups than that in the control groups.

In conclusion, although TACE plus sorafenib increases TTP in patients with HCC, it does not improve OS. Therefore, the increased risk of adverse events in patients receiving TACE plus sorafenib suggests that this combination therapy might not represent an improvement over treatment with TACE alone. The relatively small number of RCTs that met the selection criteria for our meta-analysis demonstrate the need for uniform application of clinical outcome indicators to facilitate future comparisons of studies of TACE plus sorafenib for the treatment of HCC.

## SUPPLEMENTARY MATERIALS TABLE





## References

[1] Torre LA, Bray F, Siegel RL, Ferlay J, Lortet-Tieulent J, Jemal A (2015). Global cancer statistics, 2012. CA Cancer J Clin.

[2] McGlynn KA, London WT (2011). The global epidemiology of hepatocellular carcinoma: present and future. Clin Liver Dis.

[3] Parkin DM, Bray F, Ferlay J, Pisani P (2005). Global cancer statistics, 2002. CA Cancer J Clin.

[4] Llovet JM, Burroughs A, Bruix J (2003). Hepatocellular carcinoma. Lancet.

[5] Llovet JM, Bruix J (2008). Novel advancements in the management of hepatocellular carcinoma in 2008. J Hepatol.

[6] Llovet JM, Real MI, Montaña X, Planas R, Coll S, Aponte J, Ayuso C, Sala M, Muchart J, Solà R, Rodés J, Bruix J, Barcelona Liver Cancer Group (2002). Arterial embolisation or chemoembolisation versus symptomatic treatment in patients with unresectable hepatocellular carcinoma: a randomised controlled trial. Lancet.

[7] Llovet JM, Bruix J (2003). Systematic review of randomized trials for unresectable hepatocellular carcinoma: Chemoembolization improves survival. Hepatology.

[8] Liu J, Yi J (2007). Relationship between the changes of VEGF level and dendritic cells in peripheral blood of patients with hepatocellular carcinoma after transcatheter arterial chemoembolization. J Huazhong Univ Sci Technolog Med Sci.

[9] Sergio A, Cristofori C, Cardin R, Pivetta G, Ragazzi R, Baldan A, Girardi L, Cillo U, Burra P, Giacomin A, Farinati F (2008). Transcatheter arterial chemoembolization (TACE) in hepatocellular carcinoma (HCC): the role of angiogenesis and invasiveness. Am J Gastroenterol.

[10] Wilhelm SM, Carter C, Tang L, Wilkie D, McNabola A, Rong H, Chen C, Zhang X, Vincent P, McHugh M, Cao Y, Shujath J, Gawlak S (2004). BAY 43-9006 exhibits broad spectrum oral antitumor activity and targets the RAF/MEK/ERK pathway and receptor tyrosine kinases involved in tumor progression and angiogenesis. Cancer Res.

[11] Abou-Alfa GK, Schwartz L, Ricci S, Amadori D, Santoro A, Figer A, De Greve J, Douillard JY, Lathia C, Schwartz B, Taylor I, Moscovici M, Saltz LB (2006). Phase II study of sorafenib in patients with advanced hepatocellular carcinoma. J Clin Oncol.

[12] Cheng AL, Kang YK, Chen Z, Tsao CJ, Qin S, Kim JS, Luo R, Feng J, Ye S, Yang TS, Xu J, Sun Y, Liang H (2009). Efficacy and safety of sorafenib in patients in the Asia-Pacific region with advanced hepatocellular carcinoma: a phase III randomised, double-blind, placebo-controlled trial. Lancet Oncol.

[13] Wang G, Liu Y, Zhou SF, Qiu P, Xu L, Wen P, Wen J, Xiao X (2016). Sorafenib combined with transarterial chemoembolization in patients with hepatocellular carcinoma: a meta-analysis and systematic review. Hepatol Int.

[14] Hu MD, Jia LH, Liu HB, Zhang KH, Guo GH (2016). Sorafenib in combination with transarterial chemoembolization for hepatocellular carcinoma: a meta-analysis. Eur Rev Med Pharmacol Sci.

[15] Bai W, Wang YJ, Zhao Y, Qi XS, Yin ZX, He CY, Li RJ, Wu KC, Xia JL, Fan DM, Han GH (2013). Sorafenib in combination with transarterial chemoembolization improves the survival of patients with unresectable hepatocellular carcinoma: a propensity score matching study. J Dig Dis.

[16] Muhammad A, Dhamija M, Vidyarthi G, Amodeo D, Boyd W, Miladinovic B, Kumar A (2013). Comparative effectiveness of traditional chemoembolization with or without sorafenib for hepatocellular carcinoma. World J Hepatol.

[17] Qu XD, Chen CS, Wang JH, Yan ZP, Chen JM, Gong GQ, Liu QX, Luo JJ, Liu LX, Liu R, Qian S (2012). The efficacy of TACE combined sorafenib in advanced stages hepatocellullar carcinoma. BMC Cancer.

[18] Moher D, Liberati A, Tetzlaff J, Altman DG, PRISMA Group (2009). Preferred reporting items for systematic reviews and meta-analyses: the PRISMA statement. BMJ.

[19] Lencioni R, Llovet JM, Han G, Tak WY, Yang J, Guglielmi A, Paik SW, Reig M, Kim DY, Chau GY, Luca A, Del Arbol LR, Leberre MA (2016). Sorafenib or placebo plus TACE with doxorubicin-eluting beads for intermediate stage HCC: The SPACE trial. J Hepatol.

[20] Hoffmann K, Ganten T, Gotthardtp D, Radeleff B, Settmacher U, Kollmar O, Nadalin S, Karapanagiotou-Schenkel I, von Kalle C, Jäger D, Büchler MW, Schemmer P (2015). Impact of neo-adjuvant Sorafenib treatment on liver transplantation in HCC patients - a prospective, randomized, double-blind, phase III trial. BMC Cancer.

[21] Sansonno D, Lauletta G, Russi S, Conteduca V, Sansonno L, Dammacco F (2012). Transarterial chemoembolization plus sorafenib: a sequential therapeutic scheme for HCV-related intermediate-stage hepatocellular carcinoma: a randomized clinical trial. Oncologist.

[22] Kudo M, Imanaka K, Chida N, Nakachi K, Tak WY, Takayama T, Yoon JH, Hori T, Kumada H, Hayashi N, Kaneko S, Tsubouchi H, Suh DJ (2011). Phase III study of sorafenib after transarterial chemoembolisation in Japanese and Korean patients with unresectable hepatocellular carcinoma. Eur J Cancer.

[23] Lang L (2008). FDA approves sorafenib for patients with inoperable liver cancer. Gastroenterology.

[24] Richly H, Schultheis B, Adamietz IA, Kupsch P, Grubert M, Hilger RA, Ludwig M, Brendel E, Christensen O, Strumberg D (2009). Combination of sorafenib and doxorubicin in patients with advanced hepatocellular carcinoma: results from a phase I extension trial. Eur J Cancer.

[25] Abou-Alfa GK, Johnson P, Knox JJ, Capanu M, Davidenko I, Lacava J, Leung T, Gansukh B, Saltz LB (2010). Doxorubicin plus sorafenib vs doxorubicin alone in patients with advanced hepatocellular carcinoma: a randomized trial. JAMA.

[26] Abdel-Rahman O, Elsayed ZA (2013). Combination trans arterial chemoembolization (TACE) plus sorafenib for the management of unresectable hepatocellular carcinoma: a systematic review of the literature. Dig Dis Sci.

[27] Takimoto CH, Awada A (2008). Safety and anti-tumor activity of sorafenib (Nexavar) in combination with other anti-cancer agents: a review of clinical trials. Cancer Chemother Pharmacol.

[28] Zhao Y, Li H, Bai W, Liu J, Lv W, Sahu S, Guan S, Qin X, Wang W, Ren W, Mu W, Guo W, Gu S (2016). Early sorafenib-related adverse events predict therapy response of TACE plus sorafenib: A multicenter clinical study of 606 HCC patients. Int J Cancer.

