# Supplementary material for: Transcatheter hepatic arterial chemoembolization and sorafenib for hepatocellular carcinoma: a meta-analysis of randomized, double-blind controlled trials
Source: Oncotarget. 2017 Jul 18;8(35):59601–8. doi: 10.18632/oncotarget.19334 (PMC5601759; doi:10.18632/oncotarget.19334)
Supplement: Supplementary file 1 [file oncotarget-08-59601-s001.pdf]

Transcatheter hepatic arterial chemoembolization and sorafenib for hepatocellular carcinoma: a meta-analysis of randomized, double-blind controlled trials

SUPPLEMENTARY MATERIALS

Supplementary Table 1: PRISMA checklist. See Supplementary_Table_1
